# Improving mental health care transitions for children and youth: a protocol to implement and evaluate an emergency department clinical pathway

**DOI:** 10.1186/s13012-016-0456-9

**Published:** 2016-07-07

**Authors:** Mona Jabbour, S. Reid, C. Polihronis, P. Cloutier, W. Gardner, A. Kennedy, C. Gray, R. Zemek, K. Pajer, N. Barrowman, M. Cappelli

**Affiliations:** 1 Division of Emergency Medicine, Department of Pediatrics, Children’s Hospital of Eastern Ontario, Ottawa, ON K1H 8L1 Canada; 2 Faculty of Medicine, Department of Pediatrics, University of Ottawa, Ottawa, Canada; 3 Psychiatric & Mental Health Research, CHEO Research Institute, Ottawa, Canada; 4 Department of Psychology, Carleton University, Ottawa, Canada; 5 Centre for Pediatric Mental Health Services and Policy Research, CHEO Research Institute, Ottawa, Canada; 6 Department of Epidemiology, University of Ottawa, Ottawa, Canada; 7 Psychology, Children’s Hospital of Eastern Ontario, Ottawa, Canada; 8 Department of Psychiatry, University of Ottawa, Ottawa, Canada; 9 Psychiatry, Children’s Hospital of Eastern Ontario, Ottawa, Canada; 10 Clinical Research Unit, CHEO Research Institute, Ottawa, Canada; 11 Department of Statistics, University of Ottawa, Ottawa, Canada; 12 Department of Psychology, University of Ottawa, Ottawa, Canada

**Keywords:** Clinical pathway, Mental health, Emergency department, Pediatric, Risk assessment, Service integration, Care transitions, Implementation, Scoring tools

## Abstract

**Background:**

While the emergency department (ED) is often a first point of entry for children and youth with mental health (MH) concerns, there is a limited capacity to respond to MH needs in this setting. Child MH systems are typically fragmented among multiple ministries, organizations, and providers. Communication among these groups is often poor, resulting in gaps, particularly in transitions of care, for this vulnerable population. The evidence-based Emergency Department Mental Health Clinical Pathway (EDMHCP) was created with two main goals: (1) to guide risk assessment and disposition decision-making for children and youth presenting to the ED with MH concerns and (2) to provide a streamlined transition to follow-up services with community MH agencies (CMHAs) and other providers. The purpose of this paper is to describe our study protocol to implement and evaluate the EDMHCP.

**Methods/design:**

This mixed methods health services research project will involve implementation and evaluation of the EDMHCP in four exemplar ED-CMHA dyads. The Theoretical Domains Framework will be used to develop a tailored intervention strategy to implement the EDMHCP. A multiple baseline study design and interrupted time-series analysis will be used to determine if the EDMHCP has improved health care utilization, medical management of the MH problems, and health sector coordination. The primary process outcome will be the proportion of patients with MH-specific recommendations documented in the health record. The primary service outcome will be the proportion of patients receiving the EDMHCP-recommended follow-up at 24-h or at 7 days. Data sources will include qualitative interviews, health record audits, administrative databases, and patient surveys. A concurrent process evaluation will be conducted to assess the degree of variability and fidelity in implementation across the sites.

**Discussion:**

This paper presents a novel model for measuring the effects of the EDMHCP. Our development process will identify how the EDMHCP is best implemented among partner organizations to deliver evidence-based risk management of children and youth presenting with MH concerns. More broadly, it will contribute to the body of evidence supporting clinical pathway implementation within novel partnerships.

**Trial registration:**

ClinicalTrials.gov (NCT02590302)

## Background

Approximately 80 % of children and youth with mental health (MH) problems do not receive any MH services [1–3]. However, this seems to be impacting emergency departments (EDs), which are a commonly used access point for care. Due to the scarcity of child and youth MH services, EDs have increasingly become the place that families present for help. In its baseline scorecard for child and youth MH within Ontario, Canada, the Institute for Clinical Evaluative Sciences (ICES) reported marked annual increases in ED presentations between 2009 and 2012 among youth for anxiety, mood, and substance abuse disorders [4]. At one Canadian ED, visits for MH concerns among children and youth increased, more than 50 % increase from 2010 to 2011 [5], far exceeding the 15 % increase in overall patient visits documented in the hospital administrative database.

Although families attempt to access treatment and services in these settings, EDs are ill equipped to manage these pediatric MH problems. EDs typically lack standardized MH screening tools and pediatric MH expertise. In addition, with the complex and fragmented MH systems across North America [3, 6], many EDs lack defined, reliable, and integrated referral processes to appropriate community resources. Therefore, ED clinicians have difficulty identifying the appropriate MH services to which to refer patients for follow-up care [7].

**Fig. 1:**
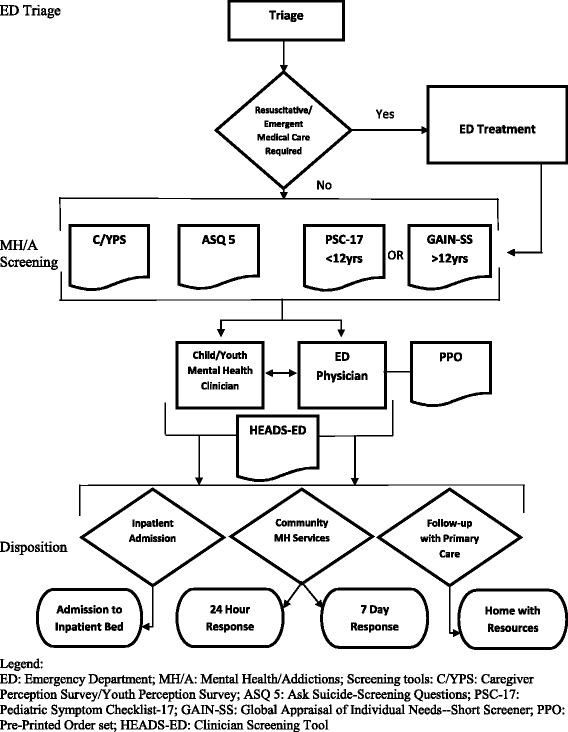
Emergency Department Mental Health Clinical Pathway algorithm

To address these concerns, a group of Canadian hospital and community-based professionals was commissioned by the Provincial Council on Maternal Child Health in Ontario to create a clinical pathway, known as the Emergency Department Mental Health Clinical Pathway (EDMHCP) for children and youth presenting with MH problems (see Fig. 1). The EDMHCP is designed to (i) improve early identification and intervention for CYMH issues, (ii) provide more timely access to community MH services, and (iii) reduce service gaps for vulnerable children and youth. It defines a standardized approach to risk assessment and disposition decision-making and while improving access to appropriate needs-based MH care [8]. Finally, the EDMHCP aims to provide a seamless transition of care for children/youth and caregivers between the ED, outpatient hospital services and community MH agencies (CMHA), with tailored linkages to community resources through the HEADS-ED screening tool [9] incorporated within the pathway.

The overall aim of our project is to implement and evaluate impact of the EDMHCP in specific settings and to evaluate the implementation process. The purpose of this paper is to document the protocol for this 3-year mixed methods health services research project. Research objectives for this study are to:

1. Implement the EDMHCP using a theory-driven, evidence-based approach. The pathway will be implemented in four dyads of organizations, each dyad comprising an ED and an associated CMHA.
2. Evaluate clinical pathway effectiveness through measurement of process and service outcomes. Our hypothesis is that a successfully implemented EDMHCP will lead to improved process and service outcomes.
3. Conduct a process evaluation of the pathway implementation strategy to assess the fidelity of the intervention delivered against the outcomes observed.

## Methods/design

The protocol for this MH project was adapted from a recent ED clinical pathway implementation protocol [10]. Collaborating with site partners, a theory-based and tailored intervention strategy will be developed to implement the EDMHCP within each ED-CMHA dyad. A concurrent process evaluation will be conducted to assess the implementation strategy. For this protocol, we completed the CONSORT 2010 checklist for randomized trials [11].

### Sampling

#### Setting and site selection

To assess EDMHCP feasibility in various settings, implementation will occur in a staggered process in four Ontario exemplar ED-CMHA dyads with different patient populations and workflows that do not currently utilize clinical pathways. Our exemplar ED sites include a pediatric specialized center with an annual patient census of 70,000, a high-volume community urban hospital (annual patient census 75,000), a low-volume rural hospital (annual patient census 25,000), and a high-volume general hospital in a smaller community (annual patient census 60,000).

#### Inclusion/exclusion criteria

To begin, commitment to the implementation intervention by an administrative lead on behalf of each hospital and CMHA will be required. The EDMHCP has specific inclusion/exclusion criteria for patients presenting to the ED, which may be modified at each site based on organizational requirements. Inclusion criteria for patient participants include (1) ages ≥6 and <18 years, (2) ED presentation within a selected 8-month implementation period, (3) MH presenting complaint, and 4) proficiency in English or French. Exclusion criteria include (1) highest priority triage designation (resuscitation), (2) medically unstable patients, (3) intubation/intensive care required, and (4) direct admission to hospital for ongoing medical management and observation. Patients who are stable but require medical management are eligible for the pathway once they are clinically able to participate in a MH assessment in the ED (see Fig. 1).

#### Participants

Participants within each study site include ED staff and physicians, CMHA clinicians, and administrators, as well as patients (and their caregivers) presenting with MH concerns. Site champions will be identified for optimal local implementation.

### Intervention strategy

**Fig. 2:**
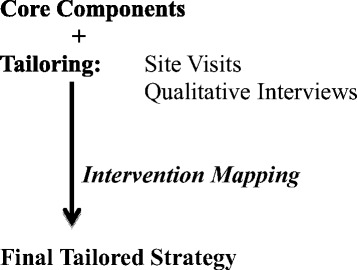
Intervention strategy

As illustrated in Fig. 2, our intervention strategy will build on core components with findings from site visits and qualitative interview findings to identify relevant behavior change techniques for our tailored intervention strategy.

#### Core components

Based on experience in related provincial and regional implementations [12–15], we have provisionally selected core intervention components, as listed in Table 1. Engagement of respected and influential champions at each site is a critical element in promoting and maintaining local interest in the pathway. Additional components include education sessions, website support, posters, and reminders. Currently, an implementation toolkit for the EDMHCP is readily available online (http://www.pcmch.on.ca/health-care-providers/paediatric-care/pcmch-strategies-and-initiatives/ed-clinical-pathways/) [8]. These instructions, measures, and training materials will be tailored for each site and hosted online during the implementation phase on the HEADS-ED website [16]. While the study team will provide guidance and ongoing support, site members will deliver relevant activities to ensure a realistic assessment of requirements for this and future implementations.

**Table 1:** Implementation strategy core components

Core component	Description
Local site champion teams	ED physician or staff (nurse educator, ED nurse)
	CMHA clinician or administrator
Hospital and CMHA Commitment	Facilitation through hospital approval processes
	Prioritization within other hospital initiatives
Site visits	Assessment of local ED culture, organization, and feedback on clinical pathway usability (human design factor analysis)
Ongoing site support	Bimonthly teleconferences with site teams
Education	Train-the-trainer workshops
	HEADS-ED training videos
Website support	Resource materials
	Study support
Posters/reminders	Clinical pathway-specific visual tools

#### Theory-based design with knowledge user input

Further aspects of our intervention strategy will be based on the Theoretical Domains Framework (TDF), which consists of a set of conceptual determinants and associated theoretical constructs that are believed to influence behavior and stimulate behavior change [17, 18]. The TDF provides a useful approach to identifying relevant behavioral determinants and designing interventions that will address these [19]. We have successfully conducted TDF-based focus groups with various urban and rural ED teams on implementing the HEADS-ED screening tool [7], an integral component of the EDMHCP. For this study, additional TDF focus groups, with up to 10 participants, will be conducted at each CMHA to elicit perceptions related to adopting the EDMHCP. Further guiding our strategy, we will assess organizational readiness and relevant factors for change through TDF-guided key informant (KI) interviews with one administrator at each site. All interviews will be audiotaped and transcribed for analysis.

#### Site visits

Implementation will begin with site visits to further assess organizational issues within CMHAs and EDs, such as intake and flow of pediatric patients, specific and shared roles of health providers, and pathway fit and acceptability. Infrastructure requirements and implementation readiness will also be assessed. A structured site visit form will be used to take notes and guide visits at each ED or CMHA.

#### Intervention mapping

We will conduct a mapping exercise to link relevant behavioral determinants among health providers identified through the qualitative interviews. We will then tailor our core strategy by applying known taxonomies of behavior change techniques [20, 21] to identify and select appropriate modes of delivery for a multifaceted intervention strategy that would go beyond simply educating for change. For example, methods may include facilitation, nudging, or systems change. Feasibility and practicality will be important considerations to ensure implementation success.

#### Ongoing support and communication

Ongoing support will be provided to expedite pathway implementation and mitigate delays due to other organizational priorities. We will communicate monthly with site champions to support progress during implementation and every 2 months in the post-implementation phase to ensure sustainability. The project coordinator will communicate regularly to ensure target dates are met, using a process log to track reasons for delays that will be explored further in the post-implementation interviews.

### Project phases

The project phases and designated timelines are outlined in Table 2. Once REB approval is secured at each site, the *pre-implementation phase* will proceed with the site readiness visits and qualitative interviews that will determine the final tailored intervention strategies. The *implementation phase* for each dyad begins with a process to secure a written memorandum agreement between the ED and CMHA to ensure clarity of responsibilities and expectations integral to the pathway. Implementation will proceed as per the tailored intervention strategy. Completed pathway implementation requires, at a minimum, the following: EDMHCP site customization and committee approvals, delivery of at least two educational workshops for each organization, and EDMHCP availability in the ED. We have designated an 8-month period for EDMHCP implementation within each dyad. Following initiation within the first dyad, subsequent dyads will begin implementation in a staggered pattern every 3 months to allow for optimal study team support and incorporating learning from experience with previous dyads. In the *post-implementation phase*, qualitative interviews will explore experience with the implementation and site audits will be done to assess implementation status. We have defined the data collection periods as follows: (i) post-implementation, as the consecutive 9-month span that follows implementation completion plus an initial 3-month “settling in” time and (ii) pre-implementation, as a similar 9-month span prior to the intervention.

**Table 2:** Description of project phases

Project phase	Duration	Activities
Pre-implementation	6 months	Site recruitment and REB approvals
		Project launch team meeting
		Site readiness visits
		Qualitative interviews and analysis
		Intervention development
		Site champion training
Implementation	17 months (8 months per dyad)	Site customization
		Forms approvals
		Clinical pathway training
		Adoption and feedback
Post-implementation	12 months	Post-implementation site visits
		Qualitative interviews and analysis
Data collection and analysis	4 months	Chart audits
		Chart to administrative database linkage
		Quantitative data analysis
Follow-up	2 months	Project partner meeting: review of findings
		Wrap-up and dissemination

### Evaluation

To assess the impact of the EDMHCP implementation and explore the factors leading to our eventual findings, we have planned a mixed methods evaluation approach employing both quantitative and qualitative evaluations, as summarized in Table 3 and described further below. Additionally, a concurrent process evaluation will be conducted to assess the degree of variability and fidelity in implementation of the intervention within each dyad. This evaluation will include a process log, post-implementation site visits, and qualitative interviews.

**Table 3:** Evaluation components

Quantitative evaluation	Qualitative evaluation
Patient chart audits	Patient surveys
Administrative data	Process evaluation Process log Post-implementation Site visits Key informant interview Focus groups
Patient surveys	

### Outcome measures

Table 4 provides a summary of study outcomes with the corresponding evaluation measures. The primary *process* outcome is the proportion of patients with documented MH-specific recommendations, as defined by the project team and based on the EDMHCP, in the medical chart. The primary MH *service* outcome is the proportion of patients that receive the EDMHCP recommended follow-up: either within 24 h or 7 days post-ED visit, based on pathway defined risk level. Recognizing logistic and scheduling factors, a successful outcome will permit a 12-h or 3-day window for follow-up. Adherence, within-window adherence, and non-adherence will be analyzed separately. Secondary outcomes include (1) EDMHCP uptake in the ED, measured as the proportion of completed clinical pathway forms filed in the health record; (2) post-ED uptake of recommended community MH services, as measured by the Services for Children and Adolescent-Parent Interview (SCA-PI) tool [21]; (3) alignment of recommended services to the HEADS-ED assessment; (4) ED length of stay, hospital admissions, ED revisits (10 days and 3 months) obtained from health records and National Ambulatory Care Reporting System (NACRS) data; and (5) patient/caregiver satisfaction with the ED visit as measured by the Services for Children and Adolescent-Parent Interview (SCA-PI) [22] and the Client Satisfaction Questionnaire (CSQ-8) [23].

**Table 4:** Study outcomes: process and service

Outcome measure		Description	Details	Data source
Process outcomes	1°	Documented MH recommendations	Proportion of patients with documented MH-specific recommendations in the medical chart	Health record audits
	2°	EDMHCP uptake in the ED	Measured as the proportion of completed CPs filed in health records	Health record audits
Service outcomes	1°	Patients receiving post-ED follow-up	Proportion of patients that receive post-ED follow-up as per EDMHCP recommendations	Health record audits
				NACRS
				Telephone follow-up
	2°	Patient perspectives of post-ED MH service uptake	Post-ED uptake of recommended community MH services	Services for Children and Adolescent-Parent Interview (SCA-PI)
		Alignment of risk assessment with MH services recommendations	Alignment of recommended services to the HEADS-ED assessment	Health record audits
				NACRS
		Hospital metrics	ED LOS, hospital admissions, ED revisits (10 days, 3 months)	Health record audits
				NACRS
		Patient satisfaction	Patient/caregiver satisfaction with the ED visit	Client Satisfaction Questionnaire (CSQ-8)

*MH* mental health, *EDMHCP* Emergency Department Mental Health Clinical Pathway, *NACRS* National Ambulatory Care Reporting System, *ED LOS* emergency department length of stay

#### Health record audits

To evaluate whether the intervention results in EDMHCP use among ED clinicians and documentation of MH-specific discharge recommendations, we will audit health records of relevant patients seen during alternate weeks in each 9-month pre- and post-implementation period. ICD10 codes (F codes, mental, and behavior disorders; X codes, intentional self-poisoning and self-harm; Y codes, poisoning and self-harm of undetermined intent) listed as primary or secondary diagnoses will identify relevant patient charts during the study period. Abstracted data will include demographic and sufficient clinical data to determine risk assessment, disposition plans, and adherence with EDMHCP recommendations. Two auditors, blinded to the study aims and protocol, will be trained to abstract and directly enter health records data into REDCap, an online database [24]. A data dictionary will be created to guide auditors and ensure standardized data collection procedures. Auditors will each abstract the same 50 charts to assess inter-rater agreement. This will be measured for key variables with a kappa coefficient and further training will be done until a prevalence and bias-adjusted kappa >0.6 is achieved.

Based on historical administrative data, the number of pediatric MH visits at the pediatric specialized center is expected to be approximately 2250 during each 9-month period. This sample size will permit estimation of a proportion at this site to within ± 2.1 %, conservatively assuming a true proportion of 50 %. At the other extreme, the lowest number of pediatric MH visits during each 9-month period among the other three sites is expected to be approximately 61 at the rural hospital, which will permit estimation of a proportion to within ±12.5 %. Pooling across sites, the total sample size in each 9-month period is anticipated to be approximately 2503, which will permit estimation of a proportion to within ± 2.0 %.

#### Administrative databases

Administrative databases will be used to evaluate whether the pathway improves health care coordination and decreases subsequent ED utilization and wait times. We will use NACRS data from each hospital to assess ED length of stay, hospital admissions, and number and frequency of repeat ED visits at 10 days and 3 months post the index ED visit. Data will be prospectively collected into a shared ED-CMHA database tool to assess CMHA service times (intake, full assessment and treatment) post-ED visit.

#### Health consumer surveys

Patient/caregiver surveys will be used to assess satisfaction with the ED visit and service within CMHAs. Because the EDMHCP recommends CMHA referral intake within 7 days, we will use structured interviews and validated measures to conduct a 10-day post-ED visit survey to assess ED visit satisfaction and whether CMHA intake has occurred. Patients or their caregivers will be contacted by e-mail, post, or phone within 7–10 days of the ED visit and will be given three follow-up measures by phone: (1) structured interview to determine if patients had initiated and received recommended MH services 10 days after being discharged from the ED; (2) the eight-item Client Satisfaction Questionnaire (CSQ-8) to determine patients’ satisfaction with heath care services received in the ED as reported by caregivers or youth; and (3) The Services for Children and Adolescent-Parent Interview (SCA-PI) which describes pediatric MH services received across multiple settings, as reported by caregivers. To reduce respondent burden, for the purpose of this study, only eight questions concerning services received for MH issues have been selected. The SCA-PI will be used to measure patients’ use of community resources and document perceptions of the ED’s role in connecting them to MH services.

### Process evaluation

To explore reasons for potential variation across in implementation effectiveness across the study sites, we will conduct a concurrent process evaluation by documenting characteristics of our intervention, as well as the participants’ response to the delivery. This evaluation will assess the degree of variability and fidelity in implementation across the sites, revealing the degree to which the intervention addressed the relevant factors for change. Specific components of this process evaluation will include process logs, data from the post-implementation site visits, and qualitative interviews. Reasons for lack of completed implementation, should this occur at any site, will be detailed and further explored in the post-implementation phase interviews. Lessons learned will be documented and shared with site partners to promote sustained pathway use.

#### Process log

A process log will be used to longitudinally track key outcomes and capture issues related to site customization of documents, barriers and delays, workshop attendance and interest, ease of use, and degree of pathway uptake. Log components will include interim survey feedback, monthly updates from site champions, and implementation progress with negotiated target dates. Additional site support will be provided as needed, based on interim findings.

#### Site visits

Two months following completed implementation, structured site visits will be conducted to assess accessibility and operational knowledge regarding the pathway. Findings will be compared to pre-implementation visits for each site.

#### Qualitative interviews

To explore organizational issues, team dynamics and other relevant issues impacting EDMHCP implementation, post-implementation interviews will be held using TDF-based interview guides. On-site focus groups will be held with up to ten participants per organization. The focus group moderator will record field note observations. As well, KI interviews will be conducted with an administrative lead for each organization. Additional focus group sessions or KI interviews will be held if data saturation is not achieved. All interview sessions will be audiotaped and transcribed for analysis.

### Analysis

#### Quantitative analysis

Descriptive statistics (means and standard deviations for continuous variables or medians with interquartile ranges) will summarize EDs, CMHAs, and patient characteristics. Frequencies and proportions for categorical variables will be reported. We will use a multiple baseline study design and conduct interrupted time-series analysis using segmented regression models, adjusting for site, with ARIMA autocorrelation structure [25–27] to evaluate whether the EDMHCP has resulted in improved health care utilization, medical management, and health sector coordination. Two-sided *p* values less than 0.05 will be considered statistically significant. Model estimates will be tabulated, together with 95 % confidence intervals. We will also calculate hospital level summary measures including proportions in the pre- and post-intervention periods at each site and referred to each CMHA. An unweighted mean of the change from pre- to post-intervention will be calculated for each hospital ED. The main effect measure will be calculated as the difference between the mean changes in intervention.

*Qualitative analysis* is integral to determine relevant factors and experience with EDMHCP use. Pre/post-site visits, focus groups, and KI interviews will yield complex data that will inform the intervention and prepare for the EDMHCP implementation (pre) and verify EDMHCP uptake status and assess stakeholders’ experiences regarding the intervention success. Interviews will be audiotaped and transcribed. Using the TDF, two coders will independently analyze all transcripts and field notes. The analyses will be provided in an overall thematic format prior to the intervention and 6-month post-intervention. To monitor progress and pursue emerging themes, data collection and analysis will proceed iteratively and concurrently [28]. The qualitative analyst will feed relevant emerging themes back to the interviewer who will revise the interview guide as needed to capture new ideas. Inductive analysis will be managed using N-Vivo10 software and will occur in three phases: coding, using the TDF [18], and categorizing/developing themes. For consistent application, codes will be operationally defined and placed into broad categories that correspond to the major units of analysis. As categories emerge, their theoretical properties will be defined. Comparisons between multiple categories will be conducted to locate similarities and differences between them. Categories will then be synthesized into themes.

### Ethics and registration

Ethics approval for this study has been granted at the coordinating site (Children’s Hospital of Eastern Ontario Research Ethics Board – CHEOREB# 15/146X). Prior to implementation within each dyad, REB approval will be obtained for each site hospital and CMHA. Informed consent will be sought for interviews and focus group meetings. All research data will be stored on a secure server. This trial is registered with ClinicalTrials.gov (NCT02590302).

### Trial status

This project is currently in the pre-implementation phase, seeking local ethics approvals and data sharing agreement, and preparing the intervention strategy.

## Discussion

By operationalizing best evidence into ED practices and formulating ED-CMHA partnerships through explicit agreements, the EDMHCP is poised to address current system gaps in addressing mental health for youth. This clinical pathway will standardize language and care provided through improved communication and explicit expectations for transition between ED and CMHA settings. Our evaluation will also rigorously address requirements for success and inform scalability to other settings. Our research will identify effective strategies to inform EDMHCP adoption in any community and provide knowledge on its impact by evaluating service integration improvements for this vulnerable population.

While trials are more typically conducted with a complete intervention strategy specified in advance, the unique nature of our proposed implementation requires rich understanding of professional and system issues within each organizational context. Hence, the importance of incorporating findings from our qualitative interviews and site visits to optimize likelihood of implementation success.

Important aspects of our tailored intervention strategy include opportunities for providers at each site to identify strengths and barriers to EDMHCP implementation and the deliberate plan to include site leads in the modification process from the beginning. The planned site visits, qualitative interviews, and continued communication will be helpful in generalizing the pathway implementation to other ED and CMHA settings of various sizes, staff, and urban/rural locations. Further, the tailored resources hosted on the HEADS-ED website [16] will be helpful reinforcement for sites that experience a high staff turnover, particularly for new ED residents and staff.

Given the complexity and novel aspects of this intervention, we anticipate some challenges in promoting modifications to current workflows. Each dyad contains the often hectic and somewhat unpredictable ED environment and the variability among CMHAs in their capacity and structure. Additionally, EDs and CMHAs do not typically interact closely, despite the shared patient population that moves between their settings. Other real-life issues such as budget restrictions, staff turnover, and competing project priorities have also been considered in setting our project timelines. At this stage, these issues are not prohibitive for any of our proposed study sites; however, this is always subject to change. Such is the challenge of implementation research.

Our study findings will be relevant for health systems and professionals responsible for ensuring standardized, quality care for children, and youth with acute MH concerns. To ensure our findings directly impact relevant service delivery areas, we have specifically recruited study team members with decision-making authority and/or influence on delivery of care for children and youth with MH concerns. We expect the protocol and findings can be customized and implemented in other EDs and CMHA settings. Equally important, this project will contribute further to implementation evidence relevant to use of clinical pathways and use of theory-based change strategies.

## Abbreviations

CHEO, Children’s Hospital of Eastern Ontario; CMHA, community mental health agency; CSQ-8, client satisfaction questionnaire; ED, emergency department; EDMHCP, Emergency Department Mental Health Clinical Pathway; HEADS-ED, screening tool that includes home, education, activities, drugs, suicidality, emotions, and discharge resources; KI, key informant (interviews); MH, mental health; NACRS, National Ambulatory Care Reporting System; REB, Research Ethics Board; SCA-PI, services for children and adolescents-parent interview; TDF, Theoretical Domains Framework
